# Let’s make it better: An updated model interpreting international student satisfaction in China based on PLS-SEM approach

**DOI:** 10.1371/journal.pone.0233546

**Published:** 2020-07-06

**Authors:** Ling Lin, Zhengwei Huang, Bestoon Othman, Yin Luo

**Affiliations:** 1 School of Foreign Languages, China Three Gorges University, Yi Chang City, Hubei Province, China; 2 China Three Gorges University, Yi Chang City, Hubei Province, China; 3 Department of Business Administration, Koya Technical Institute, Erbil Polytechnic University, Erbil, Kurdistan, Iraq; 4 Economics and Management Department, China Three Gorges University, Yi Chang City, Hubei Province, China; Univerza v Mariboru, SLOVENIA

## Abstract

Research studying into student satisfaction has been developing in the light of customer satisfaction theory which considers perceived value and quality as important predictors of student satisfaction. But the importance of value co-creation is very much overlooked. Hence this study intends to examine the relationship between perceived value, perceived quality, value co-creation, student satisfaction, complaint and loyalty to give a picture of how perceived value, perceived quality and value co-creation are important predictors of student satisfaction. Partial least square structural equation modelling is adopted to analyse responses from a survey of international students in a Chinese university. Results indicate that perceived value, perceived quality and value co-creation are determinants of student satisfaction which positively influences loyalty and negatively influences complaint. Finally, the research notes that more resources should be dedicated to engaging international students to participate in on-campus management and service work to improve international student studying experience.

## Introduction

Higher education internationalization is considered to have improved higher education quality in China. Over the past few years, Chinese higher education institutions have enrolled increasing numbers of international students. With an annual growth rate of 7.1% reported by the Ministry of Education of People’s Republic of China, China is still not considered a competitive and quality higher education service provider. However, Chinese universities are expecting to play a bigger role in the international higher education market. It thus makes sense to explore factors that might affect international student satisfaction in China.

The concept of student satisfaction resulted from an evaluation of students’ experiences was put forward by Elliot and Healy [1]. Student satisfaction was thus defined as attitude resulting from an evaluation of experiences, service and facilities. There are various developed scales to investigate student satisfaction. One is The College Student Satisfaction Scale which includes 12 to 13 indicators covering all aspects relating to American students’ college life. Another example is The College Satisfaction Scale designed by the British Institute of Higher Education focusing more on courses and learning experience. The Australian College Students Satisfaction Scale investigates how students feel about courses and campus life. The dimensions of student satisfaction include personal factors such as age, gender and preferred learning style, institutional factors such as instruction quality, instructor feedback, clarity of expectation, teaching style [2], and other factors such as classroom quality, lecturer-student relationship, interaction with fellow students, available learning equipment and facilities, library facilities and learning materials [3–5]. Student satisfaction index has been constructed based on the customer satisfaction studies. Customer satisfaction could be explained by factors such as perceived value, perceived quality, firm image, customer expectation; the consequences of customer satisfaction are customer loyalty and complaint [6]. In the light of customer satisfaction models, researchers developed structural models to account for student satisfaction [7–9]. University image, student expectation, perceived quality, perceived value are determinants of student satisfaction while loyalty and complaint are consequences.

Though there is little debate over the necessity of meeting student demands for the sake of long-term benefit on both parties of students and universities, scholars still worried about the customer-oriented view originated from marketing theory, worrying that customer focus could undermine the learning process by pandering to student [10,11]. Moreover, research has found that students feel satisfied when they are provided with quality education and when they feel a sense of belonging [12]. The gap shows the inadequacy of extant student satisfaction models in accounting for student’s happiness with their education experience. Researchers in marketing also find that customers are not just passive recipients and levels of satisfaction is linked to their efforts [13,14]. Researchers [15,16] thus proposed value co-creation as a possible factor influencing customer satisfaction. Though value co-creation has been an accepted determinant of customer satisfaction, it is not yet sufficiently explored in education context to account for student satisfaction. This study thus tries to fulfil the need by updating the student satisfaction model by integrating a value co-creation variable as possible determinant of student satisfaction.

In addition, research in student satisfaction has been most of the time conducted with domestic students. Seldom do researchers investigate into international students satisfaction. Determinants of student satisfaction might not be applicable for international students. For example, expectation is a very important factor to predict student satisfaction but fails in accounting for international student satisfaction in China. Many international students are enrolled into Chinese higher education institutions without much expectation before they land in China whose culture is quite distinctive from their home land. According to a pilot study, many international students acknowledged that they came to China because of affordable tuition and thriving Chinese economy. This implies that international student satisfaction might not be so much influenced by expectation but by what they really experience and how they value their experience process and outcome. This means extant studies of student satisfaction need to be conducted if we want to better understand what international student experience in China.

Therefore, this study aims to: (1) examine value co-creation as possible determinant of student satisfaction; (2) investigate the feasibility of upgraded conceptual model to account for international student satisfaction.

The proposed student satisfaction model includes six variables: perceived value, perceived quality, value co-creation, satisfaction, complaints and loyalty. Model reliability and validity were tested by a pilot study and then questionnaires were distributed to international students at a university in Hubei Province. To fulfil the aim, this study adopts PLS-SEM (partial least squares structural equation modelling) since the research goal is an effort of extending existing structural theory by bringing value co-creation into student satisfaction structural model [17].

The paper is organised in sections as follows: Section 1 is a brief introduction of the research. Section 2 reviews research about student satisfaction and develops the research conceptual model. Section 3 presents the research methodology. Section 4 concludes with research results, and section 5 provides discussion and policy suggestions. lastly, limitations and outlook for future research are also presented.

## Research framework and hypotheses development student satisfaction

In higher education, satisfaction occurs when students expectation is met or exceeded [18]. Delucchi [19] holds that student satisfaction is a subjective perception of higher education experience. In the light of student satisfaction models [7–9], image, expectation, perceived value, perceived quality are determinants of student satisfaction. Loyalty and complaint are consequences. Chinese researchers [20,21] investigated into domestic student satisfaction in higher education institutions by investigating variables of image, expectation, perceived quality, perceived value as determinants of student satisfaction and loyalty, complaint as consequences.

Though student satisfaction derives from assessment of education experience in terms of both process and outcomes, the extant studies of student satisfaction have been to a great extent outcome-oriented. Value co-creation has been proposed as an important contributor to customer satisfaction as marketing researchers [15,16] find that customers are rather active in assessing their consumption experience rather than passive recipients of service and products. Value co-creation has also been examined as a possible antecedent of student satisfaction [22,23]. Therefore, value co-creation is listed as a potential determinant of student satisfaction.

In an interview implemented before the official survey, international students told researchers that they came to China because of China’s thriving economy, affordable tuition and friends’ or relatives’ recommendations. University image was very blurry since they knew little about Chinese universities. With so scarce knowledge about a culturally-distinctive country, their expectation about future university life was very much ambiguous and hard to specify what to expect. Therefore, variables of university image and expectation are removed from the proposed conceptual model. Finally, a conceptual model of international student satisfaction is proposed with value co-creation, perceived value, perceived quality are antecedents of international student satisfaction with image and expectation removed; loyalty and complaint are consequences of international student satisfaction.

**Fig 1:**
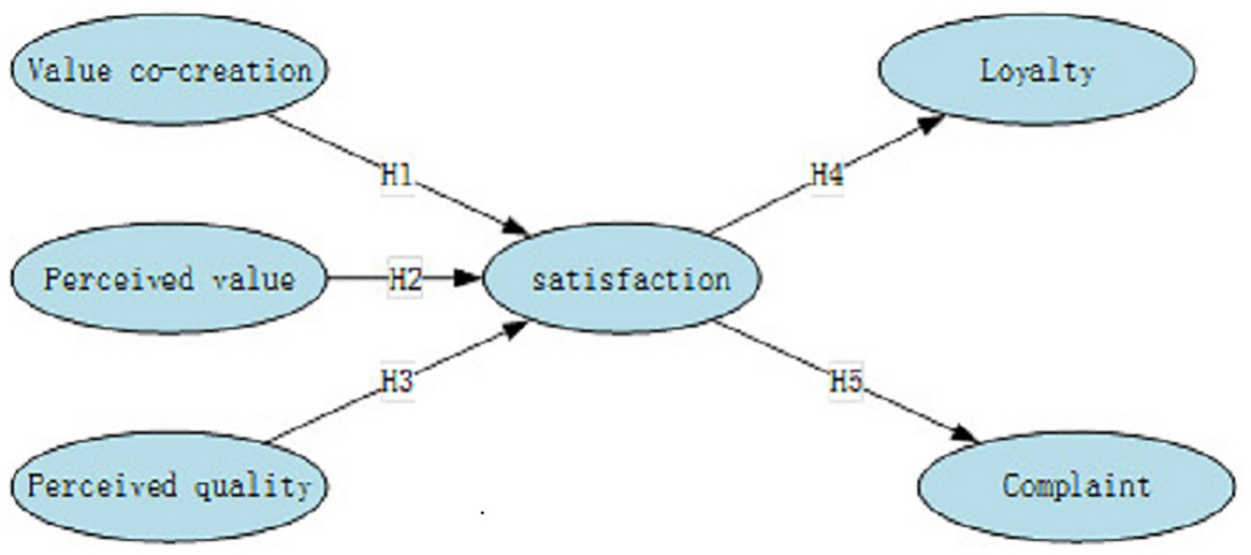
Conceptual model of international student satisfaction.

For better view, the conceptual model of international student satisfaction is displayed in Fig 1 the conceptual model of international student satisfaction.

### Value co-creation

Value co-creation is a collaborative model of marketing in which customers are an integral part of value creation [24]. In education, the value of education is created by both educators and students, with both parties playing essential roles in the value-creating process. Maxwellstuart [22] examined the relationship between support, value co-creation and student satisfaction with fee status and mode of study as moderating factors Giner and Rillo [23] empirically measured the impact of co-creation on student satisfaction and subsequent loyalty. Thus, we hypothesize:

H1: value co-creation positively influences student satisfaction.

### Perceived value

Perceived value is the difference between the total value obtained by customers and the total cost paid by customers. The more valuable customer perceives the product or service, and the lower the cost customer pays, the more likely they perceive the service or product valuable [25]. Research has shown that value perception is the major factor influencing student satisfaction in China [21]. This study examines how students perceive their education in China based on their judgement of how much they improve in terms of competency against the costs they pay or the comparison between gain and payment [26,27]. Thus, we hypothesize:

H2: perceived value positively influences student satisfaction

### Perceived quality

Perceived quality refers to customers’ feelings about the performance of products or services after purchasing. Service quality was the major factor affecting customer satisfaction [28]. For higher education, perceived quality mainly refers to the higher education services that students actually experience during their study and life after enrolment or registration. Researchers [29–31] thought that good perceived service quality of higher education has a positive impact on student satisfaction. Thus, we hypothesize:

H3: perceived quality positively influences student satisfaction.

### Loyalty

Webb and Jagun (1997) defined loyalty in the higher education context as student willingness to recommend universities to their relatives or friends, to talk positively about their educational experience and to return in pursuit of further studies [32]. Some scholars [33–35] have found a positive correlation between customer satisfaction and customer loyalty. Thus, we hypothesize:

H4: student satisfaction positively influences student loyalty.

### Complaints

Customer complaints are an expression of customer dissatisfaction with the enterprise in order to seek some form of compensation [36]. It has been found that there is a significant negative relationship between student satisfaction and complaints [21]. That is to say, the more satisfied the students are, the less likely they are to complain, or, the more satisfactory the school, the higher the student satisfaction. Thus, we hypothesize:

H5: student satisfaction negatively influences complaints.

The summary of all five hypotheses and their explanation is depicted in Table 1.

**Table 1:** Summary of hypotheses.

Hypotheses	Explanations
H1	value co-creation positively influences student satisfaction
H2	perceived value positively influences student satisfaction
H3	perceived quality positively influences student satisfaction
H4	student satisfaction positively influences student loyalty
H5	student satisfaction negatively influences complaints

To sum it up, a theoretical framework is hereby developed incorporating variables of value co-creation, perceived value, perceived quality, student satisfaction, complaint and loyalty.

To better illustrate the relationships among variables, a list of all involved variables is shown as the following.

1. Value co-creation (CC): a collaborative model of marketing in which customers are an integral part of value creation [24]
2. Perceived value (PV): difference between the total value obtained by customers and the total cost paid by customers [26,27]
3. Perceived quality (PQ): customers’ feelings about the performance of products or services after purchasing [28]
4. Student satisfaction (SA):attitude resulting from an evaluation of experiences, service and facilities [1]
5. Loyalty (LO): the extent to which customers are devoted to the product or service or how strong is the customers’ tendency to reselect the product or service. [32]
6. Complaint (CO): an expression of customer dissatisfaction with the enterprise in order to seek some form of compensation [36]

### Research methodology

#### Description of survey

Ethics Committee approval was obtained from the Institutional Ethics Committee of China Three Gorges University. This study was implemented in two-stage: a small-scale pilot survey to specify what factors might most influence international student satisfaction; formal survey. Quantitative data was collected by distributing questionnaires to international students in China Three Gorges University on December 2018 and retrieved 356 with a response rate of about 71.2%. 319 were validly completed. The questionnaire was developed based on extant research with 21 questions. Except for demographic inquiry of correspondents, variable scales were developed based on studies into student satisfaction [1,19], perceived quality [19,28], perceived value [19,26,27], value co-creation [22,23], and complaint [19,35] and loyalty [19,32]. one item for student satisfaction and one item for complaint were deleted because the statistical values didn’t quite meet the recommended value. Since 2 indicators out of 21 were deleted, with about less than 20% recommended rate. This does not undermine the validity of the model in the light of Ramayah et al. [37]. The original Chinese-written scales were then translated into English by a professional bilingual with advanced Chinese and English proficiency. After translation, an international student from America was asked to proofread the script in case of errors and ambiguity. Two versions were then compared finding no differences. For questions inquiring into variable-related items, 5-point Likert scale was used. 5-point Likert scales could be used for analytical tools like structural equation model. For the 5-point Likert scale, 1 denotes strong disagreement, 2 denotes disagreement, 3 denotes general, 4 denotes agreement, 5 denotes strong agreement. Satisfaction was also designed according to a 5-point Likert scale, where 1 denotes very dissatisfied, 2 denotes unsatisfactory, 3 denotes general, 4 denotes satisfied, 5 denotes very satisfied. The questions concerning loyalty were also designed according to a 5-point Likert scale, where 1 denotes absolutely no, 2 denotes no, 3 denotes neutral, 4 denotes yes and 5 denotes absolutely yes.

Convenience sampling was adopted for this research. With the help of workers in charge of international student affairs, questionnaires were distributed in classrooms. Students were given time to fulfil the task and questionnaires were retrieved. For this study, the response rate is 71.2% with 319 valid responses, which justified the sampling as representative of international student in China Three Gorges University [38].

The distribution of respondents by gender and degree is demonstrated in Table 2.

**Table 2:** Respondents’ information.

Information	Bachelor degree	Master degree	Doctor degree
	80.8%	2.7%	16.5%
Male total	50.3%		
Female total	49.7%		

#### Structural equation modeling

Structural equation modelling is a statistical technique for testing and estimating causal relationships and has advantages in regression and path analysis when dealing with multiple variables. Structural equation modelling has long been used as a way to implement studies in student satisfaction [21,23]. PLS-SEM was selected because it has certain advantages. Firstly, PLS-SEM could handle both formative and reflective indicators for latent variables; secondly, it requires minimum measurement scales; thirdly, it is capable of handling small size samples and data is not normally distributed. Moreover, in light of the latest research [17], PLS-SEM is the preferred SEM method when research objective is prediction. SmartPLS 3 (trial version) plus SPSS 21 were used to analyse the data collected.

## Results

### Measurement model

Since this study adopted PLS-SEM, normal distribution of data is not so much required since PLS-SEM has advantage in analysing data that is not normally distributed according to Hair et al [17,39]. Validity means the evaluation’s correctness, whether the theoretical and practical meanings are the real manifestation of the fundamental concept to be evaluated or not [37]. Three types of validity analysis are content validity, construct validity that covers convergent validity, discriminant validity and criterion validity that include reliability analysis.

#### Reliability analysis

Cronbach’s alpha coefficient was utilized in the present study along with composite reliability values to examine the inter-item consistency of the measurement items. The Cronbach’s alpha and composite reliability (CR) values should be higher than 0.70 [39]. With respect to Cronbach’s Alpha and composite reliability value, Hair [39] pointed out that the reliability which is higher than 0.9 is regarded as excellent, higher than 0.8 is fine, higher than 0.7 is adequate, higher than 0.6 is doubtful, and lower than 0.5 is substandard. Table 3 presents the values of Cronbach’s alpha and CR of all constructs. It was evident that all reliability values were higher than the recommended value of 0.70. Hence, construct reliability was confirmed.

**Table 3:** Convergent validity and measurement model.

Variable	Item	loading	Cronbach’s Alpha	Composite Reliability	Average Variance Extracted (AVE)
**Complaint**	**CO1**	**0.841**	**0.740**	**0.881**	**0.788**
	**CO2**	**0.932**			
**Loyalty**	**LO1**	**0.908**	**0.792**	**0.877**	**0.707**
	**LO2**	**0.907**			
	**LO3**	**0.688**			
**Perceived Quality**	**PQ1**	**0.751**	**0.827**	**0.879**	**0.594**
	**PQ2**	**0.855**			
	**PQ3**	**0.815**			
	**PQ4**	**0.692**			
	**PQ5**	**0.728**			
**Perceived Value**	**PV1**	**0.820**	**0.847**	**0.896**	**0.684**
	**PV2**	**0.852**			
	**PV3**	**0.874**			
	**PV4**	**0.759**			
**Student Satisfaction**	**SA1**	**0.905**	**0.797**	**0.908**	**0.831**
	**SA2**	**0.918**			
**Value Co-creation**	**CC1**	**0.708**	**0.728**	**0.844**	**0.645**
	**CC2**	**0.802**			
	**CC3**	**0.890**			

#### Convergent validity

According to Hair et al. [39], convergent validity is to assess the degree to which two measures of the same concept are correlated. They further suggest that researchers utilize the factor loadings, composite reliability (CR) and average variance extracted (AVE) to assess convergence validity. All the items loadings should be over the recommended value of 0.70 [39]. In addition, composite reliability values reflect the level to which the construct indicators reveal the latent variable and they should be greater than 0.70, as recommended by prior researchers [39]. In this study, all the composite reliability values ranged from 0.844 to 0.908, as shown in Table 2, indicating good internal consistency reliability.

On a final note, the average variance extracted (AVE) measures the variance captured by the indicators relative to measurement error and loading value higher than 0.50 was recommended to justify the use of the construct [39]. In this study, the AVEs ranged from 0.594 to 0.831, which were all within the recommended range as presented in Table 3. Therefore, the entire latent variables fulfilled the threshold value and were considered to have met the standard recommended for convergent validity.

#### Discriminant validity

*Fornell-Larcker’s criterion*. In addition to ensuring the discriminant validity of the measurement model, the current study also examined the cross loading measurement as per the indicator according to Fornell-Larcker’s criterion [40] of measurements. To determine the discriminant validity, the square root of average variance extracted (AVE) is compared against the correlations of the other constructs. Each latent variable should be larger than the latent variable correlations (LVC). As depicted in Table 4, the square root of the AVE for the variable of value co-creation, perceived value, perceived quality, student satisfaction, complaint and loyalty are much larger than the corresponding latent variable correlations. Hence, the Fornell and Larker’s criterion is achieved as shown in Table 4.

**Table 4:** Fornell and Larcker criterion.

**Variable**	**AVE**	**Complaint**	**Loyalty**	**Perceived Quality**	**Perceived Value**	**Student Satisfaction**	**Value Co-creation**
**Complaint**	**0.788**	0.887					
**Loyalty**	**0.707**	0.503	0.841				
**Perceived Quality**	**0.594**	0.474	0.660	0.770			
**Perceived Value**	**0.684**	0.453	0.601	0.638	0.827		
**Student Satisfaction**	**0.831**	0.393	0.755	0.592	0.551	0.912	
**Value Co-creation**	**0.645**	0.491	0.614	0.670	0.537	0.557	0.803

*Heterotrait-Monotrait criterion (HTMT)*. To supplement the Fornel-Lacker’s criterion [40], Henseler et al. [41] imposed a more stringent assessment of the variables’ discriminant validity by observing the heterotrait-monotrait criterion (HTMT). Henseler‘s HTMT criterion suggests that all variables are distinctively different at HTMT 0.90 cut-off point [40]. As shown in Table 5, the HTMT values for all variables are in the range from 0.495 to 0.846 and these indicate that all variables are distinctively different at values below HTMT 0.90. Importantly, the result of HTMT infers that the variables are distinctively different from one another, which also confirms the discriminant validity.

**Table 5:** Heterotrait-Monotrait ratio (HTMT).

	**Complaint**	**Loyalty**	**Perceived Quality**	**Perceived Value**	**Student Satisfaction**	**Value Co-creation**
**Complaint**						
**Loyalty**	**0.657**					
**Perceived Quality**	**0.596**	**0.806**				
**Perceived Value**	**0.570**	**0.740**	**0.759**			
**Student Satisfaction**	**0.495**	**0.821**	**0.723**	**0.658**		
**Value Co-creation**	**0.653**	**0.800**	**0.846**	**0.661**	**0.706**	

### Structural model testing

**Fig 2:**
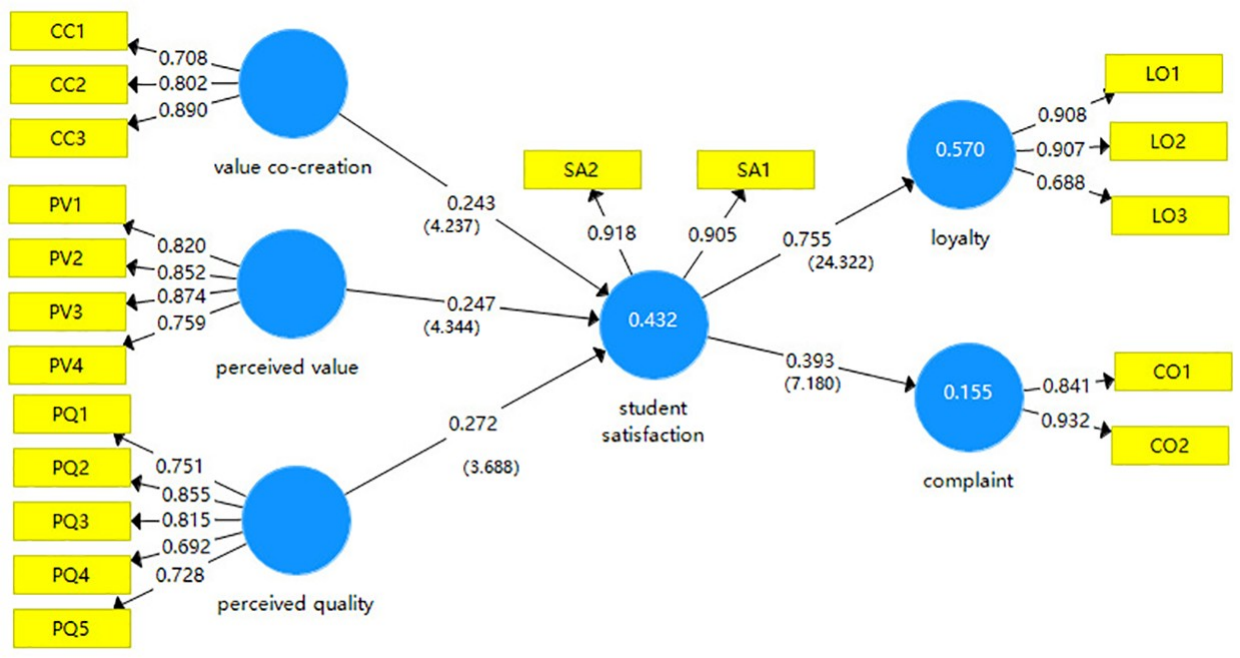
Significance of path coefficients.

Hair [17,39] thinks that R^2^ (coefficient of determination) and path coefficient and significance are most frequently reported indicators to display the global fitness of a proposed model. Though much less reported, predictive relevance (Q^2^) is also recommended by Hair [17]. R^2^ is a statistical measure of how close the data are to the fitted regression line, denoting the degree of how latent variables can be explained by manifest variables. The path coefficient between latent variables indicates the degree of variation of other variables caused by the variation of one variable. Table 6 and Fig 2 (significance of path coefficients) show R^2^, path coefficients, indicating that the model has a generally good fit. Q^2^ which is critical to assess the predictive validity of a complex model is also estimated and shown in Table 6.

**Table 6:** Predictive relevance (Q^2^).

	SSO	SSE	Q^2^ (= 1-SSE/SSO)
**Perceived Quality**	1,595.000	976.045	0.388
**Perceived Value**	1,276.000	698.717	0.452
**Value Co-creation**	957.000	662.588	0.308
**Loyalty**	957.000	561.016	0.414
**Complaint**	638.000	431.796	0.323
**Student satisfaction**	638.000	381.999	0.401

This study shows that 43.2% of the latent variable satisfaction (SA) can be explained by the exogenous variables, perceived value (PV), perceived quality (PQ), co-creation value (CC), (R^2^ = 0.432), 57% of the latent variable loyalty (LO) can be explained by satisfaction (R^2^ = 0.570), and 15.5% of the complaints (CO) can be explained by satisfaction (R^2^ = 0.155). From the variable coefficients, perceived value (PV), perceived quality (PQ), and value co-creation (CO) have a significant impact on satisfaction (SA), while satisfaction (SA) has a significant impact on loyalty (LO) and complaints (CO), among which satisfaction has a significant impact on loyalty (LO).

Table 6 shows Q^2^ values of latent variables are higher than zero, which indicates the PLS path model has predictive relevance for this construct.

Table 7 shows the relationships between the latent variables. The hypotheses concerning the relationships between variables have been well tested. t value denotes the statistical significance of the correlation between variables, the larger the T value, the higher the significance.

**Table 7:** Summary of hypotheses testing results for direct effect.

Hypotheses	Path Coefficients	Beta	Sample Mean (M)	S.E	t- value	P Values
**H1**	**PV -> SA**	0.247	0.245	0.057	4.344	**0.000**
**H2**	**PQ -> SA**	0.272	0.274	0.074	3.688	**0.000**
**H3**	**CC -> SA**	0.243	0.243	0.057	4.237	**0.000**
**H4**	**SA->LO**	0.755	0.756	0.031	24.322	**0.000**
**H5**	**SA->CO**	0.393	0.394	0.055	7.180	**0.000**

PQ = Perceived quality; SA = Student Satisfaction; PV = Perceived Value; CO = Complaint; LO = Loyalty; CC = Value Co-creation.

### Research results and comments about hypotheses

The results for Hypotheses 1, 2 and 3 show that the path coefficients of perceived value, perceived quality, value co-creation and satisfaction are 0.247, 0.272 and 0.243, respectively. International student satisfaction is positively and significantly affected by perceived value, perceived quality and value co-creation. Among them, quality perception has the greatest impact on satisfaction, followed by value perception, and finally, value co-creation. That is to say, value perception is the strongest indicator to measure foreign student satisfaction, followed by quality perception and value co-creation.

The results for Hypotheses 4 of indirect impact predicts that student satisfaction will mediate the connection perceived value, perceived quality, value co-creation and loyalty which indicates that satisfaction is the strongest predictor of international student loyalty. Finally, hypothesis 5 of indirect impact predicts that student satisfaction will mediate the connection perceived value, perceived quality, value co-creation and complaint which indicates that satisfaction is the strongest predictor of international student complaints. The more satisfied foreign students are with the educational services provided, the more loyal they are to the school. Satisfaction and complaints are positively correlated, but the index is high. That is to say, the more satisfied international students are with the school’s education service, the fewer their complaints will be, and the more view positively the university’s treatment of student complaints.

## Discussion

This study proposes a student satisfaction model consisting of perceived quality, perceived value, value co-creation, satisfaction, complaint and loyalty in the light of previous student satisfaction research. The results show that quality perception, value perception, and value co-creation have significant and positive influence on satisfaction, and that satisfaction has positive and significant influence on loyalty but negative and significant influence on complaints. Satisfaction, as a mediating variable, is influenced by quality perception, value perception and value co-creation and proves to be an indicator of international students’ loyalty and complaints. In particular, value co-creation leads to international student satisfaction as much as perceived quality and value.

The study contributes in two aspects. Theoretically speaking, it again highlights the view that a student’s perception of education experience is to a great extent both process and outcome oriented. Therefore, it is sensible to develop student satisfaction model by integrating value co-creation as an influential determinant of the student satisfaction. moreover, this model is validated to be applicable to international students. Practically speaking, this study has validated the proposed model, implying that educational practitioners in higher education institutions are strongly recommended to spend efforts in providing quality education as much as in providing value co-creation opportunities. Greater student involvement in university management and development could lead to higher level of student satisfaction which in turn results in loyalty increase. A strong sense of belonging and involvement in an organization development benefit both students and educational institutions in the long run.

In addition, this study also sheds light on international student education in China. International education has been flourishing in China with influx of international students getting enrolled. Nevertheless, previous investigations into international student satisfaction inform us that international students are generally happy with their education in China though with some complaints [42, 43]. Complaints from international students in China focused on teaching quality and campus management. Inefficient communication between international students and faculty has to a certain extent undermines course quality because of language problem. In addition, previous interviews also indicate that international students are willing to get involved into campus management work and student service. Conventionally Chinse higher education institutions treat international students as subjects they should serve, help and manage. The finding implies that providing more on-campus job vacancies could help to create and strengthen a sense of belonging.

## Limitations and outlook for future research

This study has some limitations. The student satisfaction model was proposed and validated with international students. Further research needs to be conducted to validate the model with domestic students. In addition, data was collected from a local State-owned university, which might influence the generalizability of the proposed student satisfaction model. As for future research, value co-creation is defined in the light of service marketing theory, which still needs to be investigated in the context of higher education. Previous research [44] shows that value co-creation is often defined in two dimensions in service marketing: co-production and value in-use. Research into conceptual elements of value co-creation and measurement is required.

## Supporting information

S1 File Variables and indicators. (DOCX)

S2 File Data for international students. (CSV)

## References

[1] Elliott K M & Healy M A. Key factors influencing student satisfaction related to recruitment and retention. Journal of Marketing for Higher Education, 2001; 10(4):p.1–11.

[2] Appletonknapp S L & Krentler K A. Measuring Student Expectations and Their Effects on Satisfaction: The Importance of Managing Student Expectations. Journal of Marketing Education, 2006; 28(3):p.254–264.

[3] Garciaaracil A. European graduates’ level of satisfaction with higher education. Higher Education, 2009, 57(1):p.1–21.

[4] Kuh G D, Hu S. The Effects of Student-Faculty Interaction In the 1990s. The Review of Higher Education, 2001; 24(3):p.309–332.

[5] Sojkin B, Bartkowiak P, Skuza A, et al Determinants of higher education choices and student satisfaction: the case of Poland. Higher Education, 2012; 63(5):p.565–581.

[6] Johnson MD, Gustafsson A, Andreassen TW, Lervik L, Cha J. The evolution and future of national customer satisfaction index models. Journal of Economic Psychology. 2001; 22: p. 217–245.

[7] Duque L C, Weeks J R. Towards a model and methodology for assessing student learning outcomes and satisfaction. Quality Assurance in Education, 2010; 18(2):p. 84–105.

[8] Gibson A. Measuring business student satisfaction: A review and summary of the major predictors. Journal of Higher Education Policy and Managements, 2010; 32(3):p.251–259.

[9] Karna S, Julin P. A framework for measuring student and staff satisfaction with university campus facilities. Quality Assurance in Education, 2015;23(1);p. 47–66.

[10] Bay D, Daniel H. The student is not the customer: An alternative perspective. Journal of Marketing for Higher Education, 2001;11(1);p.1–19.

[11] Buck G H. The customer is always right. Alberta Journal of Educational Research, 2002;48(1);p.1–4.

[12] Elliott K M. Key Determinants of Student Satisfaction. Journal of College Student Retention: Research, Theory and Practice, 2002, 4(3):p.271–279.

[13] Hill F M. Managing service quality in higher education: The role of the student as primary consumer. Quality Assurance in Education, 1995; 3(3);p.10–21.

[14] Lengnickhall C A. Customer contributions to quality: A different view of the customer-oriented firm. Academy of Management Review, 1996;21(3);p.791–824.

[15] Vegavazquez M, Revillacamacho M, Cossiosilva F J, et al The value co-creation process as a determinant of customer satisfaction. Management Decision, 2013; 51(10):p.1945–1953.

[16] Cossiosilva F, Revillacamacho M, Vegavazquez M, Palaciosflorencio B. Value co-creation and customer loyalty. Journal of Business Research, 2016; 69(5):p.1621–1625.

[17] Hair J F, Hollingsworth C L, Randolph A B, Chong A Y. An updated and expanded assessment of PLS-SEM in information systems research. Industrial Management and Data Systems, 2017; 117(3):p.442–458.

[18] Elliott K M, Shin D. Student Satisfaction: An alternative approach to assessing this important concept. Journal of Higher Education Policy and Management, 2002; 24(2):p.197–209.

[19] Delucchi M. Student satisfaction with higher education during 1970s’: A decade of social change. The Edwin Mellen Press, United Kingdom; 2003.

[20] Liu W, Yang X. On customer satisfaction measurement in higher education evaluation. Journal of Public Administration, 2005; (04);p.34–49.

[21] Liu H. A PLS-SEM-based Study on the Satisfaction Evaluation of Higher Education in China. (Doctor Dissertation) Jiangsu University, Suzhou, Jiangsu Province, China. 2010.

[22] Maxwellstuart R, Taheri B, Paterson A, Ogorman K D, & Jackson W J. Working together to increase student satisfaction: exploring the effects of mode of study and fee status. Studies in Higher Education, 2018; 43(8):p.1392–1404.

[23] Giner G R, Rillo A P. Structural equation modeling of co-creation and its influence on the student’s satisfaction and loyalty towards university. Journal of Computational and Applied Mathematics, 2016; 291(1):p.257–263.

[24] Mane A G, Diop P A. Drivers of customer brand engagement and value co-creation in China: A prioritization approach. International Journal of Management Science and Business Administration, 2017; 3(4):p.7–19.

[25] SaÂnchez-FernaÂndez R, Iniesta-Bonillo MA. Consumer perception of value: Literature review and a new conceptual framework. Journal of Consumer Satisfaction, Dissatisfaction and Complaining Behavior, 2006; 19: p. 40–58.

[26] Cronin JJ, Brady M, Hult GT. Assessing the effects of quality, value and customer satisfaction on consumer behavioral intentions in service environments. Journal of Retailing, 2000; 76: p. 193–218.

[27] McDougall GHG, Levesque T. Customer satisfaction with services: Putting perceived value into the equation. Journal of Services Marketing, 2000; 14:p. 392–410.

[28] Parasuraman A, Zeithaml VA, Berry LL. A conceptual model of service quality and its implication s for future research. The Journal of Marketing,1985;49:p. 41–50.

[29] Farahmandian S, Minavand H, Afshardost M. Perceived service quality and student satisfaction in higher education. IOSR Journal of Business and Management, 2013; 12(4):p.65–74.

[30] Annamdevula S, Bellamkonda R S. Effect of student perceived service quality on student satisfaction, loyalty and motivation in Indian universities. Journal of Modelling in Management, 2016; 11(2):p.488–517.

[31] Alves H, Raposo M. Conceptual model of student satisfaction in higher education. Total Quality Management & Business Excellence, 2007;18(5):p. 571–588.

[32] Webb D, Jagun A. Customer care, customer satisfaction, value, loyalty and complaining behavior: validation in a UK university setting. Journal of Consumer Satisfaction, Dissatisfaction and Complaining Behavior.1997;1:p.139–151

[33] Ali F, Zhou Y, Hussain K, Nair P K, Ragavan N A. Does higher education service quality effect student satisfaction, image and loyalty? Quality Assurance in Education, 2016; 24(1):p.70–94.

[34] Brown R M, Mazzarol T. The importance of institutional image to student satisfaction and loyalty within higher education. Higher Education, 2009; 58(1):p. 81–95.

[35] Shahsavar T, Sudzina F. Student satisfaction and loyalty in Denmark: Application of EPSI methodology. PLoS ONE, 2017;12(12). 10.1371/journal.pone.0189576 PMC5730189 29240801

[36] Maxham J. G., & Netemeyer R. G. Modelling customer perceptions of complaint handling over time: the effects of perceived justice on satisfaction and intent. Journal of Retailing, 2002; 78(4), 239–252.

[37] Ramayah T, Cheah J, Chuah F, Ting H, Memon M A. Partial least squares structural equation modeling using SmartPLS 3.0-an updated and practical guide to statistical analysis. Kuala Lumpur, Malaysia: Pearson 2018.

[38] Krejcie R V & Morgan D W. Determining sample size for research activities. Educational and psychological Measurement, 1970; 30(3), 607–610.

[39] Hair J F, Ringle C M, Sarstedt M. PLS-SEM: indeed a silver bullet. The Journal of Marketing Theory and Practice, 2011; 19(2):p.139–152.

[40] Fornell C, Larcker D F. Evaluating structural equation models with unobservable variables and measurement error. Journal of Marketing Research, 1981;18(1):p. 39–50.

[41] Henseler J, Ringle C M, Sarstedt M. A new criterion for assessing discriminant validity in variance-based structural equation modeling. Journal of the Academy of Marketing Science, 2015; 43(1):p.115–135.

[42] Wen W, Chen L, Bai Y, Cao Hs. A comparative study of international students’ experiences and satisfaction in Beijing. Social Sciences of Beijing, 2013; 2:p.63–70.

[43] Tian Y, He L M, Wang W. A comparative study of the college student satisfaction of America, Australia, Canada and the UK. Shanghai Journal of Educational Evaluation, 2016;6:32–36.

[44] Ranjan K R, Read S. Value co-creation: concept and measurement. Journal of the Academy of Marketing Science, 2016; 44(3): 290–315.

