# Correction: Let’s make it better: An updated model interpreting international student satisfaction in China based on PLS-SEM approach

**DOI:** 10.1371/journal.pone.0242583

**Published:** 2020-11-12

**Authors:** Ling Lin, Zhengwei Huang, Bestoon Othman, Yin Luo

Information is missing in the Funding statement. The correct Funding statement is as follows: This research was supported by Hubei Provintial Department of Education in the form of a grant awarded to LL (2018GA027) and China Three Gorges University in the form of a grant awarded to LL (SDKC201928). The funders had no role in study design, data collection and analysis, decision to publish, or preparation of the manuscript.

There are errors in the author affiliations. The correct affiliations are as follows:

Ling Lin^1,2^, Zhengwei Huang ^3^, Bestoon Othman^4^, Luo Yin^3^

**1** School of Foreign Languages, Department, China Three Gorges University, Yichang City, Hubei Province, China, **2** School of Foreign Languages, Hubei University of Education, Wuhan, China, **3** Economics and Management Department, China Three Gorges University, Yichang City, Hubei Province, China, **4** Department of Business Administration, Koya Technical Institute, Erbil Polytechnic University, Iraq and Scientific Research and Development Center-Nawroz University-Kurdistan Regional, Iraq
