# Supplementary material for: Let’s make it better: An updated model interpreting international student satisfaction in China based on PLS-SEM approach
Source: PLoS One. 2020 Jul 6;15(7):e0233546. doi: 10.1371/journal.pone.0233546 (PMC7337283; doi:10.1371/journal.pone.0233546)
Supplement: S1 File — (DOCX) [file pone.0233546.s001.docx]

Table 1: summary of dimensions and indicators for nominated constructs

| constructs | dimensions | Indicators |
| --- | --- | --- |
| Perceived value  PV | PV1 | I learned knowledge. |
|  | PV2 | I learned how to work in teams. |
|  | PV3 | I think my creativity has developed here. |
|  | PV4 | I developed my social skills |
| Perceived quality  PQ | PQ1 | The courses here are very valuable. |
|  | PQ2 | The teachers here are very knowledgeable and professional. |
|  | PQ3 | The classroom design here is good and helpful to my study. |
|  | PQ4 | The teacher helped a lot and was very professional. |
|  | PQ5 | The teachers here are helpful for my personal and career development |
| Value co-creation  CC | CC1 | I participated in the student service provided by the school. |
|  | CC2 | Together with the school, I tried to solve the difficulties in my studies and life. |
|  | CC3 | The university encourages us to participate in campus life and management. |
| Student satisfaction  SA | SA1 | Would you choose this school again? |
|  | SA2 | You are now generally satisfied with your study and life here as follows: |
| Loyalty  LO | LO1 | I would recommend this school to others. |
|  | LO2 | If I continue my studies, I would consider attending this school. |
|  | LO3 | In the future, if I have the ability, I would donate money to this school. |
| Complaint  CO | CO1 | I am basically satisfied with the handling of complaints by the school. |
|  | CO2 | I have complained/I believe my complaint has been properly handled. |
